# X-ray Tomographic Method to Study the Internal Structure of a TiNi–TiB_2_ Metal Matrix Composite Obtained by Direct Laser Deposition

**DOI:** 10.3390/ma16041353

**Published:** 2023-02-05

**Authors:** Maxim Korobenkov, Mikhail Lebedev, Vladimir Promakhov, Anton Narikovich

**Affiliations:** 1 International Research Center “Coherent X-ray Optics for Megascience Facilities”, Immanuel Kant Baltic Federal University, Alexander Nevsky Str., 14, 236016 Kaliningrad, Russia; 2 Scientific and Educational Center “Additive Technologies”, National Research Tomsk State University, Lenin Avenue, 36, 634050 Tomsk, Russia

**Keywords:** X-ray computed tomography, metal–ceramic composite, additive manufacturing, direct laser deposition, structure

## Abstract

The field of additive manufacturing (AM) of various materials is rapidly developing. At the stage of designing and growing products and for the quality control of finished parts, non-destructive methods of analysis, in particular X-ray computed tomography (CT), are in demand. In addition to the advantages of non-destructive imaging of a wide range of materials in three dimensions, modern CT scanners offer a high contrast and high spatial resolution to provide digital information about their three-dimensional geometry and properties. Within the framework of this article, CT was used to follow the structural evolution of a TiNi–TiB_2_ metal–ceramic composite obtained by direct laser deposition. The relationship has been established between the additive method of production (layered direct laser deposition) and the formed layered structure of the product in the direction of growth. The porosity of the sample was calculated for each scan direction, and the average for the sample was 1.96%. The matrix of the TiNi–TiB_2_ composite is characterized by the presence of pores of various sizes, shapes and locations. Spherical voids prevail, but keyhole pores are also found. The heterogeneity of the structure was revealed in the form of clearly traced boundaries of the print layers, as well as differences in the density of the inner and outer regions of the composite.

## 1. Introduction

Over the past few decades, the additive manufacturing of various materials, also known as 3D printing or rapid prototyping, has experienced phenomenal growth in its commercial, scientific and engineering development. Three-dimensional printing not only simplifies and accelerates the production of many materials, it also allows the creation of composites of unique shapes, structures and complex geometries that could not be obtained by conventional methods. For any additive manufacturing (AM) material, characterizing the internal structure in 3D is crucial for determining the macroscopic properties. To date, microstructural information for the quality control of finished parts at the design and growth stages of the product is still a big challenge.

One of the most common methods of examining the internal structure of materials is non-destructive testing. Non-destructive testing means testing a sample without causing any damage. This allows the studied samples to be used for further experimental research. Out of the many non-destructive testing methods, one of the most advanced is X-ray computed tomography (CT) [1], which provides a clear characterization of the internal defects in additively manufactured materials. The ability to scan objects in 3D without slicing or sectioning is the biggest advantage of X-ray CT over other microscopy techniques, such as scanning electron microscopy, transmission electron microscopy and optical microscopy. X-ray CT has been used to study various 3D printed materials in terms of size, spatial distribution and the morphology of defects [2,3,4,5,6,7,8].

The principle of X-ray CT is based on the absorption of X-rays, which results in 3D imaging of various materials with a resolution in the micron range. The visualization of the three-dimensional structure carried out by building a model based on a software implementation of mathematical algorithms for reconstructing projection data [9,10,11]. X-ray CT has been used to characterize the microstructure and properties of a wide range of materials, such as geological materials (rocks, soils and fossils) [12,13,14], metals and alloys [15,16,17], ceramic composites [18,19,20], polymers [21,22] and construction materials [23,24,25]. The most promising application of this method is the analysis of composite materials composed of several heterogenetic phases, which are mixed in order to obtain a material with a set property characteristic for each separate component.

In this work, we look at a composite with a metal matrix. Metal-additive manufacturing, especially by laser powder sintering, is increasingly being used for the series manufacturing of critical components in the aerospace, automotive and other industries. The usage of lasers allows various metal alloys with excellent structural integrity and mechanical properties to be produced, sometimes superior to the traditionally produced equivalent materials [26]. However, despite all the advantages of additive manufacturing, quality control is necessary to improve processes and refine the technology for parts’ production. In addition to the information obtained by standard methods—measuring density, porosity and pore size distribution—X-ray CT allows the visualization of the composite’s structure in large volumes and for detailed investigation, including of various phases, interfaces, pores, cracks and other defects. The possibility of the non-destructive quantitative determination of characteristics, and following the comparison of the data obtained from mechanical tests, mean it is possible to gain an idea of the defects’ influence on the characteristics of the producing parts [27]. This makes improving the laser deposition technology (the powder feed rate, laser power and scanning speed, laser spot size and printed layer size, etc.) possible to obtain defect-free structures.

This technology is not only used to obtain completely metal products. Composites based on a metal matrix, more specifically metal ceramics, are of great interest. These complex materials have the properties of both metals and ceramics. Their high strength and sufficiently high plasticity, wear and heat resistance and anti-corrosion properties allow for the expansion of the sphere of application and durability of traditional parts. Among modern metal–ceramic composites, materials based on a metal matrix with shape memory have an important place. Such materials with a composition based on a titanium nickelide (TiNi) intermetallic compound and the reinforcing particle titanium diboride (TiB_2_) are the investigated in this article [28,29,30]. The ceramic component is one of the most durable materials and widely used to make cutting tools for metal work. Its hardness (25–30 GPa), high melting temperature and increased resistance to wear abrasion contribute to its successful usage as a reinforcing filler in metal matrixes [31,32]. The main difficulty in creating this metal–ceramic material is that the synthesized samples are not amenable to mechanical processing due to their high strength. Therefore, the usage of the additive technology of direct laser deposition from a previously prepared NiTi–TiB_2_ powder with spherical particles is viable [28,29,30]. In addition, the proposed method allows the control of the technological process by changing the composition of the powder mixture in the growing process, achieving partial melting of the powder.

However, there are many unsolved challenges. During laser fusing of the powder mixture, pores are formed, which reduce the products’ fatigue characteristics [1,33,34,35]. It is believed that the pores act as crack initiators under cyclic loading, which may cause premature failure. It is probably the main cause of widespread fatigue properties [26]. In addition, the melting technology’s optimal parameters have not yet been established, in which the products would have a monolithic and homogeneous structure [36]. To examine the set tasks, the three-dimensional visual presentation of the internal structure of composites obtained by 3D printing is necessary, which will give more information for the research being carried out, saving money and time without destroying the finished sample. For this purpose, X-ray computed tomography is used in this work.

## 2. Materials and Methods

**Figure 1:**
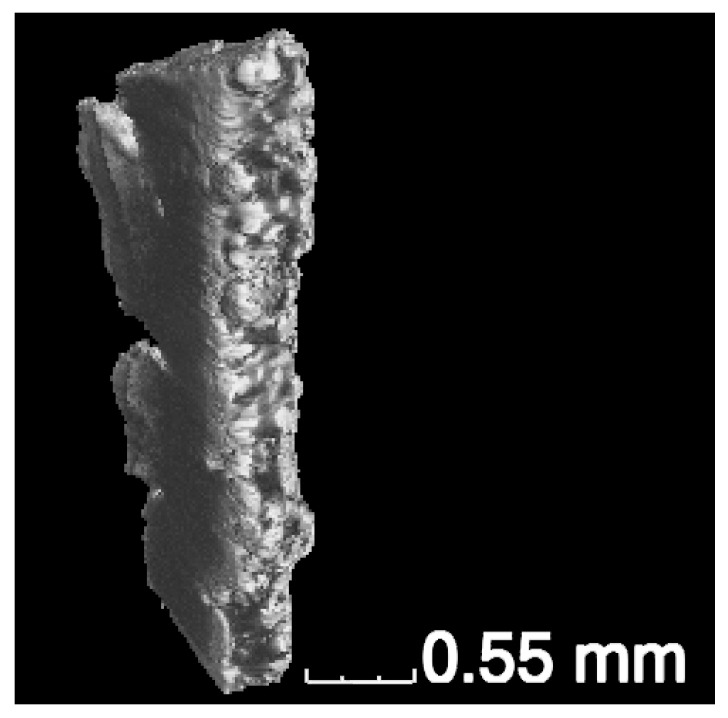
Three-dimensional image of the TiNi–TiB_2_ metal–matrix composite obtained by direct laser deposition.

The research sample used a composite material obtained by an additive method—direct laser deposition—from the powder of the TiNi intermetallic compound including of ceramic microparticles TiB_2_. The preliminarily prepared NiTi–TiB_2_ raw material mixture had a particle size of 50–150 µm; the content of titanium diboride had an average crystallite size of 180 nm and 65% [28,29,30]. The metal–matrix composite sample was an elongated irregular parallelepiped shape, at a size of 1.36 mm × 0.69 mm × 4.06 mm (Figure 1).

**Figure 2:**
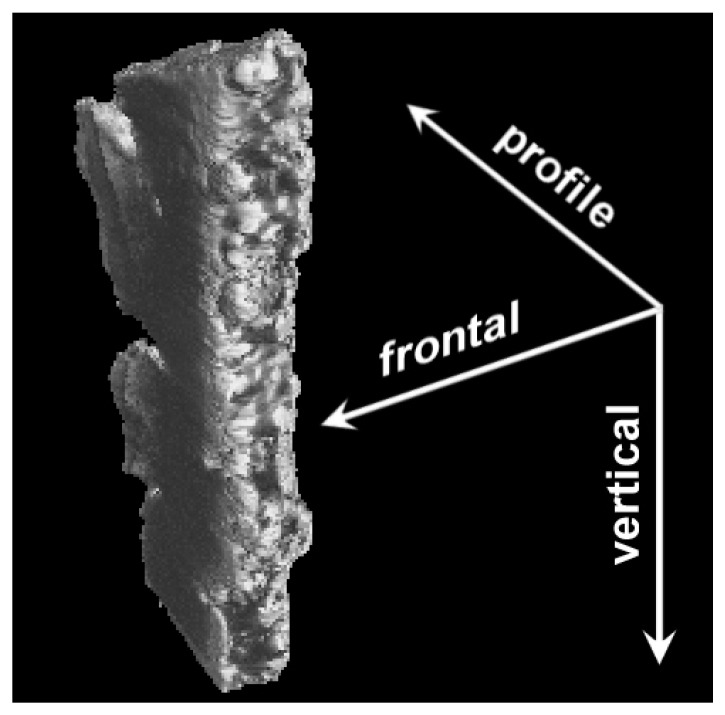
The sample’s scanning directions by X-ray tomography.

The metal–ceramic sample was investigated by X-ray computed tomography using an X-ray control system with Y. CHEETAH tomography function of the YXLON company (Hamburg, Germany), which is part of the instrumental base of the International Research Center Coherent X-ray optics for Megascience facilities of the Immanuel Kant Baltic Federal University. Two-dimensional cross-sectional images were made in three mutually perpendicular directions (Figure 2). Accordingly, three types of projections were obtained: frontal projections with frontal scanning; profile projections with profile scanning; and horizontal projections with vertical scanning. The scan step was 4.2 μm. Due to the anisotropy of the sample, the space resolution (voxel) was different: in frontal scanning it was 4.2 μm, in profile scanning it was 3.6 μm and in vertical scanning it was 1.5 μm. The 3D reconstruction of the sample structure was made by using the VGStudio MAX 2.2 software (by Volume Graphics (Heidelberg, Germany)).

**Figure 3:**
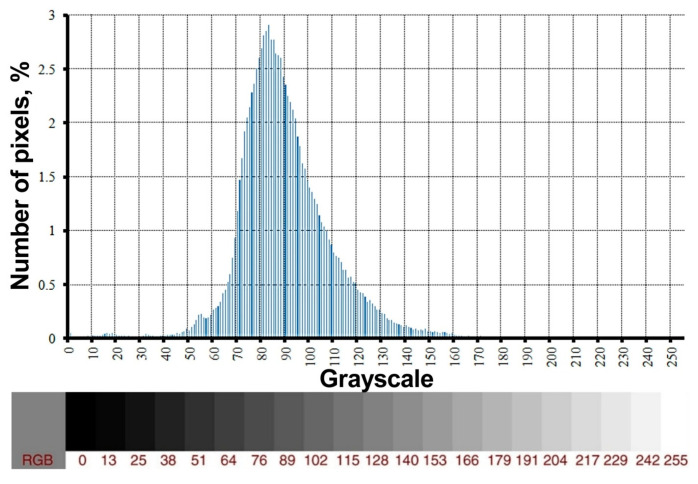
Distribution of the X-ray absorption coefficient in a form of grayscale values in the sample section obtained by the method of X-ray tomography.

The cross-sectional images of the sample obtained during the scan were processed and analyzed in order to obtain the quantitative data. The black background in the images was removed using the magic wand tool in the Corel Photo-Paint™ 2021 program. A distribution of the X-ray absorption coefficient was recorded in grayscale values (from 0 (black) to 255 (white)) (Figure 3). The measurement of the gray color intensity along a straight line and analysis of the distribution of shades over the area was performed using the ImageJ ver.1.53 program.

**Figure 4:**
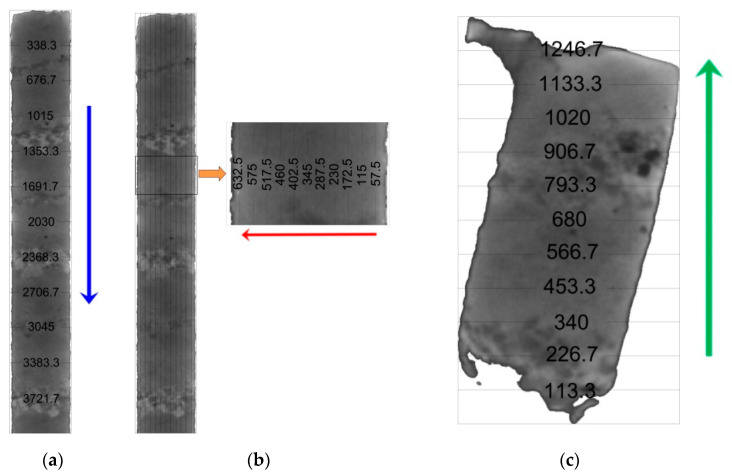
The location of the sections selected for analysis in the scanning directions of the sample (arrows indicate the scanning direction, numbers—the distance from the zero point of the sample (a scan beginning), in μm). (**a**) Vertical scanning; (**b**) frontal scanning; (**c**) profile scanning.

To analyze the distribution of structural elements (matrix, pores, inhomogeneities, etc.) throughout the sample, 11 cross-sections equidistant from neighboring sections were selected for each scanning direction (Figure 4). Due to the sample’s anisotropy, the selection of the cross-sections was different: for vertical scanning it was 338.3 μm, for frontal scanning it was 57.5 μm and with profile scanning it was 113.3 μm.

**Figure 5:**
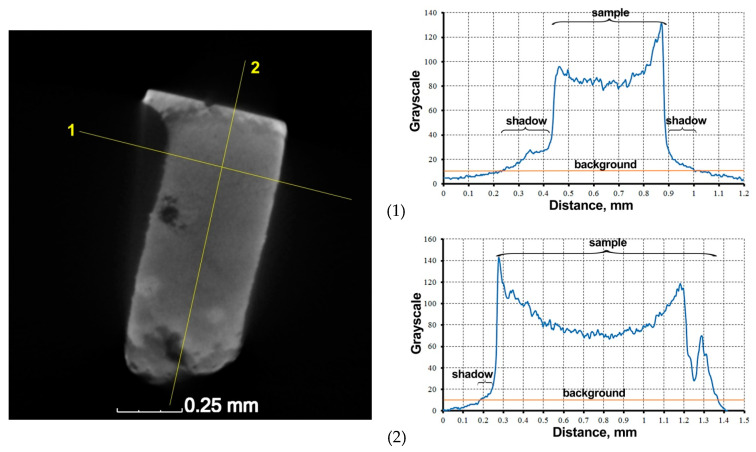
The projection of the sample with the expressed “shadow” from the underlaying surfaces.

The images obtained as a result of scanning are characterized not only by the presence of the black background, but also the so-called “shadow” from the underlaying surfaces of the sample (Figure 5).

**Figure 6:**
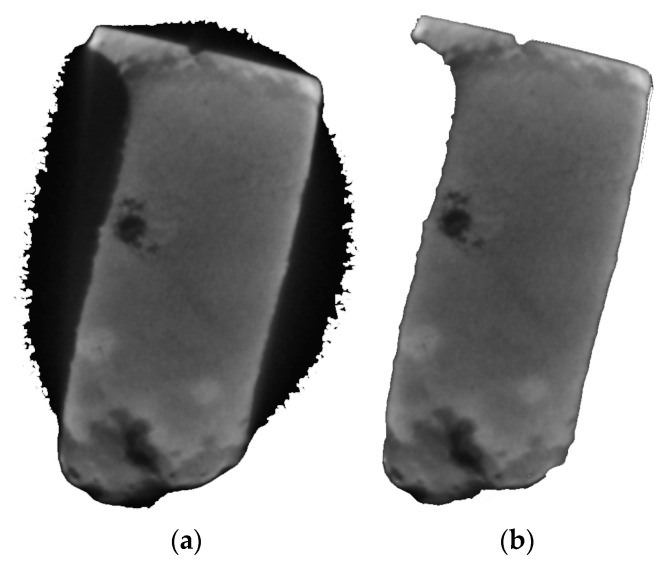
The image of the horizontal projection of the sample after background subtraction (**a**) and the “shadow” subtraction (**b**).

To obtain the correct distribution of the absorption coefficient over the section area of the sample, in addition to the background, the “shadow” was also removed; this does not lie in the analyzed projection plane (Figure 6).

## 3. Results and Discussion

The X-ray CT image is a map of the space distribution of the X-ray absorption coefficient. In this map, brighter areas correspond to higher coefficient values and more solid areas of the material. X-ray computed tomography obtains the images that display the differences in the density at each point on two-dimensional sections throughout the sample. Therefore, it is possible to visualize the details within the sample, because the absorption coefficient at each point directly depends on the sample density at that point.

**Figure 7:**
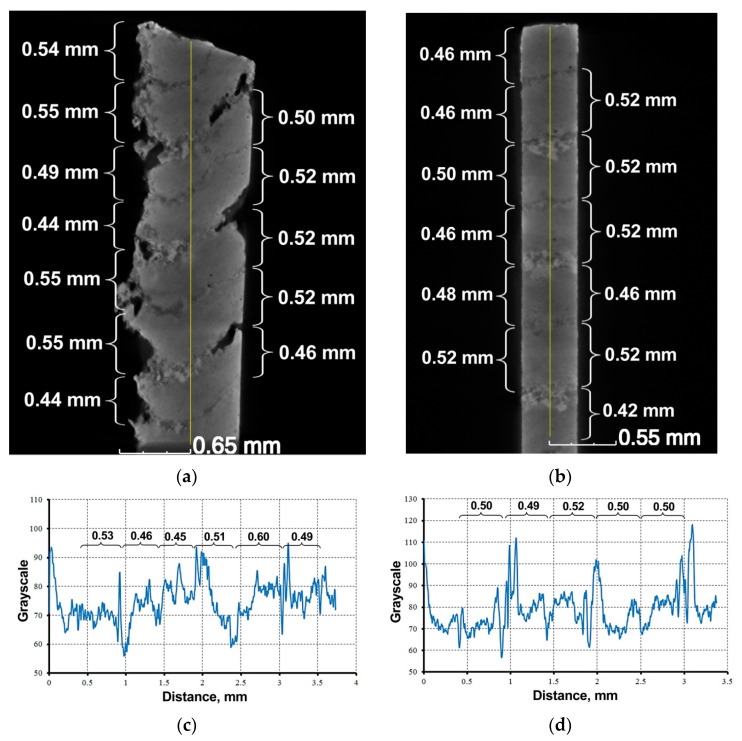
Frontal (**a**) and profile (**b**) projections of the TiNi–TiB_2_ metal–matrix composite obtained by direct laser deposition, and the corresponding graphical representation of the change in gray values along the vertical line of the section (**c**,**d**).

The investigated metal–matrix composite TiNi–TiB_2_ was obtained by direct laser deposition, layer by layer. Accordingly, the sample has a layer structure, which can be seen on the vertical projections of the X-ray CT (Figure 7). According to [28,29], the vertical shift step was 0.6 mm. The obtained images show the layer’s thickness is slightly less and is approximately 0.42–0.55 mm (Figure 7). This is a result of the powder partially melting and filling the pore space between the spherical particles of the original powder mixture. The layer’s height is not constant in the vertical or horizontal direction (Figure 7a). A layer’s slope on the horizontal plane was visible, which may be a feature of 3D printing.

On the vertical projections, the boundaries of the layers were clearly seen. Moreover, a clear alternation of dark and light border zones was observed, i.e., less dense and more dense, respectively. This can be explained by the irregular melting of the NiTi–TiB_2_ powder during the additive manufacturing of the sample. The structure formed could be a result of the fact that in the applied method of heterophase laser growth, it occurs through the incomplete melting of particles, which ensures the absence of a melt pool [28]. Figure 7c,d show the changes in grayscale values along the vertical lines of the sections in Figure 7a,b. The thicknesses of the layers shown by curly arrows in Figure 7c,d correlate with the real images of the sections in Figure 7a,b. Then, after comparison, the analysis was carried out, and it was found that the intermediate areas between the horizontal boundaries of the layers were characterized by sufficient homogeneity, which is expressed in small variations in grayscale values from 70 to 80 (in the vast majority of cases). Grayscale values below 70 and above 80 characterize the boundaries of the layers, and in Figure 7c,d they appear in the form of extrema for the most part. The smallest value is determined by the porosity of this area and the largest by the presence of denser particles with an increased fraction of TiNi intermetallide in the composition. The stronger edges of the inner regions of the metal matrix composite TiNi–TiB_2_ were also observed to be less dense compared to the outer.

**Figure 8:**
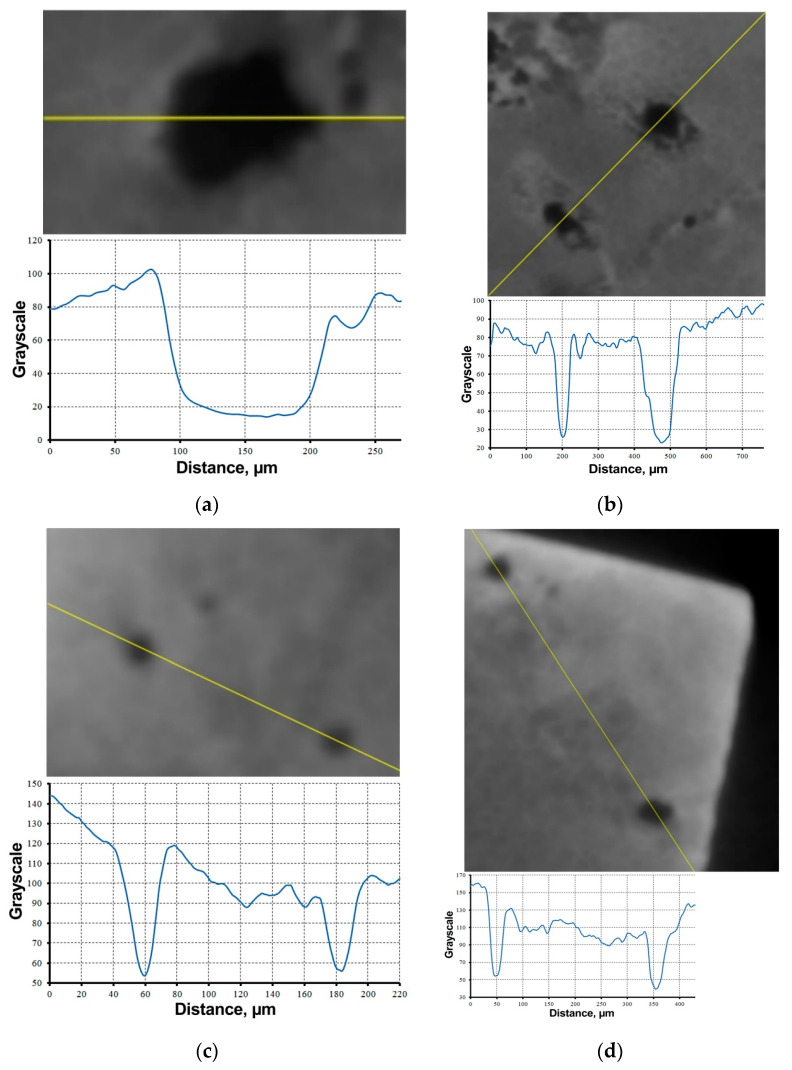
Pores in the TiNi–TiB_2_ metal–matrix composite obtained by direct laser deposition, and a graphical representation of the change in grayscale values along the cutting plane line in the plane of the pore location—inside pores (**a**–**c**), surface pores (**d**).

The matrix of the TiNi–TiB_2_ composite is characterized by the presence of pores (Figure 8). As can be seen from the images, the pores have different sizes, shapes and locations—they can be located both deep inside the sample (Figure 8a–c) and close to the surface (Figure 8d), and they can be located both within the layer and in the interlayer zone (Figure 7). It was noticed that in the X-ray CT images, the larger the pore size, the lower the absorption coefficient, and vice versa. For example, for pores with a diameter of approximately 120 µm (Figure 8a), the grayscale value was a minimum of 15–20. Smaller 30–40 µm pores (Figure 8d) are characterized by the minimum grayscale value of 40–50. Pores 20–30 µm in diameter (Figure 8c) have minimum color depth of 50–55. The increase in the grayscale value with decreasing pore size can be related to the “shadow” created within it from the underlying surfaces of the sample. This “shadow” will appear stronger and have a higher grayscale value with a smaller the pore diameter, assuming the pore shape is spherical. Conversely, the larger the pore, the farther away the underlying surface of the sample will be.

On two-dimensional projections, the pores are usually round, which suggests that they are spherical in the bulk of the composite. However, there are pores in the shape of a “keyhole” (Figure 8b). Lumley explains that pores this shape are formed by low speeds of laser growth and the trapping of metal vapors in a deeply penetrating melt pool [37]. Some researchers point to a laser power that is too high as the reason for the formation of spherical pores, while low levels lead to the formation of irregular voids due to incomplete melting [34,35,38].

To calculate the porosity value of the TiNi–TiB_2_ investigated sample, it is necessary to set the upper threshold value for the shade of gray, which would describe the presence of voids in the composite matrix. As noted previously, small pores are characterized by minimum color value of 50–55 (Figure 8c), and the sample matrix body is 70–80 units (Figure 7c,d). On this basis, the grayscale value of 60 units was chosen as the upper pore limit. Further, for all the selected cross-sections, the X-ray absorption coefficient distribution in the form of grayscale values was obtained, and the porosity value and the average value of the distribution of grayscale values were calculated (Table 1).

**Figure 9:**
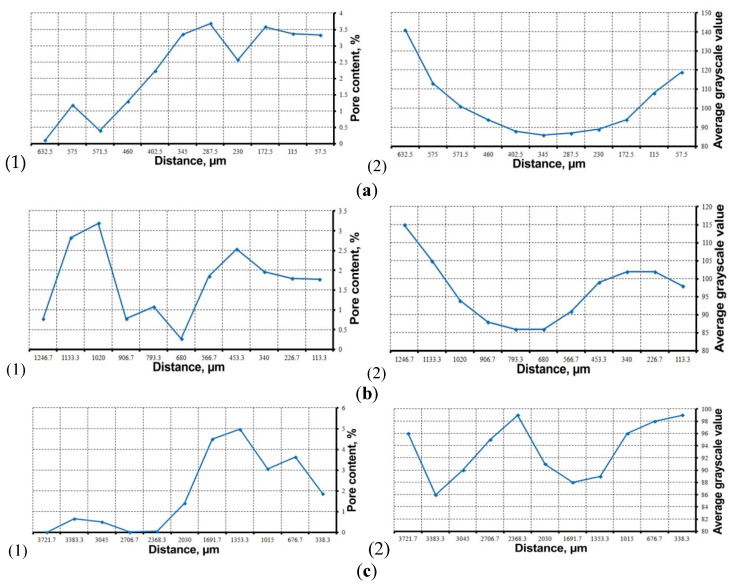
Change in porosity (**1**) and average grayscale value (**2**) along the scan direction: (**a**) frontal, (**b**) profile, (**c**) vertical.

According to the obtained data, the calculated porosity, averaged over all selected sections of each CT scan direction, fluctuates in a small range from 1.71% to 2.28% and averages 1.96% for the sample. However, the large values of the standard deviation and the coefficients of variation (58, 54 and 99%, respectively) stand out. This is due to the distribution of non-regular pores along each scan direction, as seen in Figure 9. The pore content varies from 0 to 3.7% during scanning in the horizontal direction (Figure 9a,b), in the vertical direction it ranges from 0 to 5% (Figure 9c). Moreover, the obtained curves of porosity changes are probably governed by a definite dependence, which can be explained by the parameters and features of 3D printing.

In addition, the change in the average grayscale value for each of the sections was estimated, which is an indirect characteristic of the matrix density. Here the dependence is more obvious—the inner regions of the sample are darker, and the outer ones are lighter (Figure 9). Moreover, this can be said for any scan direction—during vertical scanning the cross-sections 2368.3 µm, 2706.7 µm and 3721.7 µm penetrate the light interlayer regions (Figure 9c). Thus, the inner regions of the TiNi–TiB_2_ metal–matrix composite are less dense than the outer ones. It can be assumed that in the process of laser growth, the less-dense ceramic TiB_2_ phase (ρ = 4.52 g/cm^3^) is redistributed through the melt into the inner regions, and the peripheral areas are enriched with the denser TiNi intermetallic (ρ = 6.45 g/cm^3^). In addition, for the outer regions, the oxidation of surfaces with the formation of titanium and nickel oxides is possible, and in the central zone the formation of nano- and micropores (the space resolution of the obtained CT images is from 1.5 to 4.2 μm) due to strong evaporation, which promotes the structure loosening and the decrease in the X-ray absorption coefficient.

## 4. Conclusions

The inner structure of the TiNi–TiB_2_ metal–ceramic composite was obtained by direct laser deposition from spherical powder particles of the same composition, and was investigated by X-ray computed tomography. The exposure of two-dimensional cross-sectional images and analysis to obtain quantitative data was carried out in three mutually perpendicular directions.

The relationship was established between the additive method of production and the formed layered structure of the product in a vertical projection—the direction of growth. The measured layer height was 0.42–0.55 mm, which is less than the planned 0.6 mm. This is a result of the powder partially melting and filling the pore space between the spherical particles of the original powder mixture.

The heterogeneity of the structure is established for the distribution of the X-ray absorption coefficient in the vertical direction. The clear alternation of dark and light boundaries of the layers is observed, i.e., less dense and more dense, respectively. This can be explained by the irregular melting of the NiTi–TiB_2_ powder during the additive manufacturing of the sample. In addition, the layer’s slope to the horizontal plane is visible, which may be a feature of 3D printing. The intermediate areas between the horizontal boundaries of the layers are characterized by sufficient homogeneity.

The matrix of the TiNi–TiB_2_ composite is characterized by the presence of pores of various sizes, shapes and locations—they can be located both deep inside the sample and close to the surface or they can be located both within the layer and in the interlayer zone. The lower limit of the pore size is limited by the space resolution of the computed tomography method; the largest pores are larger than 100 microns. Spherical voids prevail, but keyhole pores are also found, the appearance of which is explained by low speeds of laser growth and the trapping of metal vapors in a deeply penetrating melt pool.

The porosity of the composite was calculated for each direction of the CT scan. It fluctuates in a small range from 1.71% to 2.28% and averages 1.96% for the sample. The non-regular pore distribution along each scan direction was revealed: in the horizontal direction from 0 to 3.7%, in the vertical direction from 0 to 5%.

It was found that the inner regions of the TiNi–TiB_2_ metal–matrix composite are less dense than the outer ones. It can be assumed that in the process of laser growth, the less-dense ceramic TiB_2_ phase (ρ = 4.52 g/cm^3^) is redistributed through the melt into the inner regions, and the peripheral areas are enriched with the denser TiNi intermetallic (ρ = 6.45 g/cm^3^).

Information on the inner structure of the TiNi–TiB_2_ metal–ceramic composite obtained by X-ray computed tomography will be used in further work to optimize the parameters of the powder-deposition technology. It is possible to obtain products with more monolithic and homogeneous structures with the additive method.

## Figures and Tables

**Table 1:** Porosity and mean grayscale value for X-ray CT projections.

Projection Type	Average Value of Pore Content, %	Standard Deviation	Average Grayscale Value	Standard Deviation
Frontal	2.28	1.33	102	17
Profile	1.71	0.92	97	9
Horizontal	1.89	1.87	93	5
